# Occupational lifestyle diseases: An emerging issue

**DOI:** 10.4103/0019-5278.58912

**Published:** 2009-12

**Authors:** Mukesh Sharma, P. K. Majumdar

**Affiliations:** Occupational Medicine Division, National Institute of Occupational Health (NIOH), Meghani Nagar, Ahmedabad - 380 016, India

**Keywords:** Chronic liver diseases, hypertension, occupational health

## Abstract

Lifestyle diseases characterize those diseases whose occurrence is primarily based on the daily habits of people and are a result of an inappropriate relationship of people with their environment. The main factors contributing to lifestyle diseases include bad food habits, physical inactivity, wrong body posture, and disturbed biological clock. A report, jointly prepared by the World Health Organization (WHO) and the World Economic Forum, says India will incur an accumulated loss of $236.6 billion by 2015 on account of unhealthy lifestyles and faulty diet. According to the report, 60% of all deaths worldwide in 2005 (35 million) resulted from noncommunicable diseases and accounted for 44% of premature deaths. What's worse, around 80% of these deaths will occur in low and middle-income countries like India which are also crippled by an ever increasing burden of infectious diseases, poor maternal and perinatal conditions and nutritional deficiencies. According to a survey conducted by the Associated Chamber of Commerce and Industry (ASSOC-HAM), 68% of working women in the age bracket of 21-52 years were found to be afflicted with lifestyle ailments such as obesity, depression, chronic backache, diabetes and hypertension. The study ‘Preventive Healthcare and Corporate Female Workforce’ also said that long hours and working under strict deadlines cause up to 75% of working women to suffer from depression or general anxiety disorder, compared to women with lesser levels of psychological demand at work. The study cited scientific evidence that healthy diet and adequate physical activity - at least 30 minutes of moderate activity at least five days a week - helped prevent NCDs. In India, 10% of adults suffer from hypertension while the country is home to 25-30 million diabetics. Three out of every 1,000 people suffer a stroke. The number of deaths due to heart attack is projected to increase from 1.2 million to 2 million in 2010. The diet [or lifestyle] of different populations might partly determine their rates of cancer, and the basis for this hypothesis was strengthened by results of studies showing that people who migrate from one country to another generally acquire the cancer rates of the new host country, suggesting that environmental [or lifestyle factors] rather than genetic factors are the key determinants of the international variation in cancer rates. Some of the common diseases encountered because of occupational lifestyle are Alzheimer's disease, arteriosclerosis, cancer, chronic liver disease/cirrhosis, chronic obstructive pulmonary disease (COPD), diabetes, hypertension, heart disease, nephritis/CRF, and stroke. Occupational lifestyle diseases include those caused by the factors present in the vicinity like heat, sound, dust, fumes, smoke, cold, and other pollutants. These factors are responsible for allergy, respiratory and hearing problems, and heat or cold shock. So, A healthy lifestyle must be adopted to combat these diseases with a proper balanced diet, physical activity and by giving due respect to biological clock. Kids spending too much time slouched in front of the TV or PCs, should be encourage to find a physical sport or activity they enjoy. Fun exercises should be encouraged into family outings. A pizza-and-video evening should be replaced for a hike and picnic. Kids who do participate in sport, especially at a high competitive level, can find the pressure to succeed very stressful. To decrease the ailments caused by occupational postures, one should avoid long sitting hours and should take frequent breaks for stretching or for other works involving physical movements.

## INTRODUCTION

People are predisposed to various diseases based on their way of living and occupational habits. They are preventable, and can be lowered with changes in diet, lifestyle, and environment. Lifestyle diseases characterize those diseases whose occurrence is primarily based on daily habits of people and are a result of an inappropriate relationship of people with their environment. The onset of these lifestyle diseases is insidious, they take years to develop, and once encountered do not lend themselves easily to cure. The main factors contributing to the lifestyle diseases include bad food habits, physical inactivity, wrong body posture, and disturbed biological clock. The diet [or lifestyle] of different populations might partly determine their rates of cancer, and the basis for this hypothesis was strengthened by results of studies showing that people who migrate from one country to another generally acquire the cancer rates of the new host country, suggesting that environmental [or lifestyle factors] rather than genetic factors are the key determinants of the international variation in cancer rates.[1–3]

A report, jointly prepared by the World Health Organization and the World Economic Forum,[4] says India will incur an accumulated loss of $236.6 billion by 2015 on account of unhealthy lifestyles and faulty diet. The resultant chronic diseases - heart disease, stroke, cancer, diabetes and respiratory infections - which are ailments of long duration and slow progression, will severely affect people's earnings. The income loss to Indians because of these diseases, which was $8.7 billion in 2005, is projected to rise to $54 billion in 2015. Pakistan woul d face an accumulated loss of $30.7 billion with income loss increasing by $5.5 billion to $6.7 billion by 2015. China, however, will be worse off. While its accumulated loss will stand at $557.7 billion, the loss of income of the Chinese will stand at $131.8 billion, almost eight times what it was in 2005.

According to the report, 60% of all deaths worldwide in 2005 (35 million) resulted from noncommunicable diseases and accounted for 44% of premature deaths. What's worse, around 80% of these deaths will occur in low and middle-income countries like India which are also crippled by an ever increasing burden of infectious diseases, poor maternal and perinatal conditions and nutritional deficiencies. Almost half of those who die from chronic diseases will be in their productive years. The report also points to the fact that countries like Brazil, China, Russia and India currently lose more than 20 million productive life-years annually to chronic diseases. And the number is expected to grow by 65% by 2030. In 2007, nearly 3.1 billion people were economically active. The figure is estimated to exceed 3.6 billion in 2020. The cost to employers of morbidity attributed to non-communicable diseases is increasingly rapidly. Workplaces should make possible healthy food choices and support physical activity. Unhealthy diets and excessive energy intake, physical inactivity and tobacco use are major risk factors for non-communicable diseases, the report said.[4]

According to a survey conducted by the Associated Chamber of Commerce and Industry (ASSOCHAM),[5] 68% of working women in the age bracket of 21-52 years were found to be afflicted with lifestyle ailments such as obesity, depression, chronic backache, diabetes and hypertension.

The study ‘Preventive Healthcare and Corporate Female Workforce’[6] also said that long hours and working under strict deadlines cause up to 75% of working women to suffer from depression or general anxiety disorder, compared to women with lesser levels of psychological demand at work. Women employed in sectors that demand more time such as media, knowledge process outsourcing and touring jobs are unable to take leave when unwell, and force themselves to work mainly due to job insecurity, especially during the current financial meltdown, the report said.

However, it said, factors such exposure to industrial pollutants and environmental toxins, poor quality of sleep, lack of exercise, sunlight exposure, poor nutrition, excessive intake of alcohol etc might play a confounding role and are the priority areas for further research. Highlighting the fact that women play vital and multiple roles, especially those employed, the report stressed on the need for a balance at home and workplace. “Ignorance of healthcare can have multiple implications on her surrounding environment such as her family, workplace and social network,” said the study. Over 77% of respondents said they avoided routine check-ups,” the report stated, indicating that the hectic schedule of balancing workplace and home, along with balancing between social and personal requirements lead to women ignoring their health. The report further stated that 47% of respondents spent less than Rs. 500 on healthcare in a year, while 22% spent in the range of Rs. 500-Rs. 5,000 as they suffered ailments such as obesity, depression and spondylosis. Over 29% respondents were found to be spending between Rs. 5,000-50,000 on healthcare annually. However, most of these respondents were found to be afflicted with high or low blood pressure, diabetes, heart diseases, asthma, urinary infection and arthritis.

The study cited scientific evidence that healthy diet and adequate physical activity, at least 30 minutes of moderate activity at least five days a week, helped prevent NCDs. In India, 10% of adults suffer from hypertension while the country is home to 25-30 million diabetics. Three out of every 1,000 people suffer a stroke. The number of deaths due to heart attack is projected to increase from 1.2 million to 2 million in 2010.

## DISCUSSION

According to a research paper published in the prestigious Lancet[7] there is corroborative evidence that diet and lifestyle is playing a major role in predisposition to various diseases like cancer. In many countries, peoples' diet changed substantially in the second half of the twentieth century with increase in consumption of meat, dairy products, vegetable oils, fruit juice, and alcoholic beverages, and decrease in consumption of starchy staple foods such as bread, potatoes, rice, and maize flour. Other aspects of lifestyle also changed, notably, large reductions in physical activity and prevalence of obesity.

In the 1970s it was noted that people in many western countries had diets high in animal products, fat, and sugar, and high rates of cancer of the colorectum, breast, prostate, endometrium, and lung; by contrast, individuals in developing countries usually had diet which were based on one or two starchy staple foods, with low intakes of animal products, fat, and sugar, and low rates of these cancers. These observations suggest that the diet [or lifestyle] of different populations might partly determine their rates of cancer, and the basis for this hypothesis was strengthened by results of studies showing that people who migrate from one country to another generally acquire the cancer rates of the new host country, suggesting that environmental [or lifestyle factors] rather than genetic factors are the key determinants of the international variation in cancer rates.

With advancement of our lifestyle we have become dependent on technology and gadgets which directly have an impact on our health. In a study conducted by Dr. Masayuki Tatemichi, Toho University School of Medicine,[8] heavy computer use could be linked to glaucoma, especially among those who are short-sighted. Glaucoma is caused by increased fluid pressure within the eye compressing the nerves at the back, which can lead to blindness if not treated. Workers classified as heavy computer users were more likely to be long-sighted (hypermetropia) or short-sighted (myopia). Around a third (165) of these workers had suspected glaucoma. Upon further analysis, heavy computer use, suspected glaucoma and short-sightedness appeared to be interlinked. Regular spending of lot of time in front of computer may lead to neck and back pain because body is going to begin to change and adapt to take on this frequent activity.

The front neck muscles will slowly grow shorter and tighter, while the muscles in the back of the neck will grow longer and weaker. The stiffening of neck also is a common problem along with headache, fatigue and exhaustion. Wrong sitting or standing posture while working gives strain to the backbone and gives a chronic back pain. The heat generated by laptops kept on the lap of males cause decrease in sperm count. Other extensively used gadget is mobile phone which is supposed to be a culprit for a number of diseases and ailments, although its adverse effects on humans are yet to be established and validated. Research conducted on animals in different countries has established a number of adverse effects of mobile phone radiation like low fertility, reduction in attention and memory, increase in reaction time, leakage in blood-brain barrier, sleep disturbances, headache, ringing in the ears, hearing disturbances etc.

People working in night shifts witness a disturbed biological clock leading to insomnia, indigestion, acidity, loss of appetite, headache, irritability, hypertension, mood fluctuations and body pain. Those having late night parties also experience the same with some additional effects of untimely munching, drinking and smoking. Alteration in the circadian rhythm of a person compromises his immunity, further leading to various opportunistic diseases.

Traffic on the Indian roads is also responsible for many common ailments for frequent travelers or those who earn their living by driving. According to the research study bad public transportation and faulty office postures rides for quite a long time can lead to a chronic back pain. Research also shows that most of the (41%) people suffering from backache are users of public transport and spend two to five hours in front of the computers in Delhi. Bumpy ride shakes our body too much and the impact generated is so high that it damages the vertebra with time. It may cause spinal cord injury, which is still incurable. Many people develop spondylolysis, which generally occurs due to breaking down (dissolution) of a part of vertebra, leading to localized back pain. People dealing with huge weights are predisposed to slip disk and sciatica.

Occupational lifestyle diseases include those caused by the factors present in the vicinity like heat, sound, dust, fumes, smoke, cold, and other pollutants. These factors are responsible for allergy, respiratory and hearing problems, and heat or cold shock. Those occupations, in which there is a huge temperature or pressure difference, cause a disturbed homeostasis leading to disease. Astronauts and divers have to spend time in a closed box to minimize the risk developed by the pressure difference. Similarly people working in high temperatures face problems related to BP, metabolism, and organ failure due to shock. Extreme cold working condition causes hypothermia and shock. People in the fishing or shipping industry face seasickness or motion sickness along with other risk factors contributing to a diseased condition.

All the contributing factors have significant effect on various diseases alone as well as in combination with other factors. Some of the common diseases encountered because of occupational lifestyle are Alzheimer's disease, arteriosclerosis, cancer, chronic liver disease/cirrhosis, chronic obstructive pulmonary disease (COPD), diabetes, hypertension, heart disease, nephritis/CRF, and stroke.

A studyconducted on students from government and public schools in Delhi from November 2005 showed that 26% of the kids aged 14-17 years had Syndrome X (caused by obesity), a precursor to diabetes; 1,168 students from 15 schools were screened - obesity was found to be the major cause of other problems.[9] Children who grow up to be obese could suffer from diabetes, stroke, liver diseases, infertility, hypertension, arthritis and cancer. Obese children also have a high risk of development of early heart diseases since 13-25% of them showed high levels of C-reactive protein, 40-50% had increased triglycerides (blood fat) levels and 30-70% had low HDL (good cholesterol) levels. In the screening, it was found that the level of obesity was higher in public school students than the government school students. Twenty eight per cent of urban children had Syndrome X, one step away from diabetes and two steps away from heart disease and junk food aggravates the problem. The results show an alarming trend of obesity and related diseases.

## CONCLUSIONS

The western lifestyle, characterized by convenience food, TV and PCs, is taking its toll on children as well as adults, and is producing increased numbers of overweight, passive youngsters with lifestyle diseases.[10–18] Kids spending too much time slouched in front of the TV or PCs, should be encouraged to find a physical sport or activity they enjoy. Fun exercises should be encouraged into family outings. A pizza-and-video evening should be replaced for a hike and picnic. Kids who do participate in sport, especially at a high competitive level, can find the pressure to succeed very stressful. It's important that parents watch out for signs of psychological strain, as well as physical fatigue from overtraining. Young athletes also have specific nutritional needs that require extra attention. A diet of only junk food, overeating and lack of physical activity are not only responsible for diseases related to nutrition, but also anorexia nervosa, which involves many people starving themselves for maintaining their figure. This type of disease is more prevalent in the fashion and showbiz industry.

A healthy lifestyle must be adopted to combat these diseases with a proper balanced diet, physical activity and by giving due respect to biological clock. To decrease the ailments caused by occupational postures, one should avoid long sitting hours and should take frequent breaks for stretching or for other works involving physical movements. An ergonomic chair should be designed based on the human contour to fit the right sitting posture so that the uneven pressure on joints and muscles may be minimized. In this revolutionized era we cannot stop doing the developmental work, but we can certainly reduce our ailments by incorporating these simple and effective measures to our lives.
